# Possibility of hypoalbuminemia itself as a risk for clinical failure

**DOI:** 10.1128/aac.00367-24

**Published:** 2024-04-17

**Authors:** Shougen Sumiyoshi, Shungo Yamamoto

**Affiliations:** 1 Division of Infection Control and Prevention, Osaka University Hospital, Osaka, Japan; 2 Department of Transformative Infection Control Development Studies, Osaka University Graduate School of Medicine, Osaka, Japan; 3 Division of Fostering Required Medical Human Resources, Center for Infectious Disease Education and Research (CiDER), Osaka University, Osaka, Japan; Providence Portland Medical Center, Portland, Oregon, USA

**Keywords:** ceftriaxone, hypoalbuminemia, obesity

## LETTER

We appreciate the efforts of Arensman Hannan et al. in conducting an interesting study (1). They reported that patients with hypoalbuminemia were associated with a lower proportion of clinical success among patients with obesity who were treated with ceftriaxone. They concluded that the presence of hypoalbuminemia may result in suboptimal drug exposure because ceftriaxone is a highly protein-bound antibiotic.

Our primary concern is that the patient group with hypoalbuminemia may be at a higher risk of clinical failure regardless of ceftriaxone use, as inferred from published data (2, 3).

Arensman Hannan et al. applied propensity score (PS) weighting, which revealed that there were no significant differences between the two groups in terms of demographics, the Charlson Comorbidity Index, the need for ICU admission, and the indication for ceftriaxone use, as evidenced by the absence of significant *P* values. However, because of the small sample size, it would be more appropriate to compare differences between groups using the standardized mean difference instead of *P* values (4, 5). According to the data they presented, the proportion of male and bacteremia was numerically higher, even after PS weighting, and these imbalances may have negatively influenced the hypoalbuminemia group.

Additionally, the proportion of bacteria detection from sterile specimens was 76.5% (26 of 34) in the hypoalbuminemia group compared to 62.1% (64 of 103) in the normal albumin group. Since this is a retrospective study, the lower percentage of bacteria identified in sterile specimens in the normal albumin group may have caused this group to include more noninfectious conditions than the hypoalbuminemia group. This may have resulted in an apparently better prognosis for the normal albumin group.

Therefore, we cannot rule out the possibility that hypoalbuminemia itself led to a poor prognosis in this design.

If the presence of hypoalbuminemia does indeed indicate an increased risk of clinical failure with ceftriaxone, we propose comparing ceftriaxone with an antibiotic that has a lower protein-binding affinity and a comparable spectrum, such as cefotaxime, using only the patient group with hypoalbuminemia.

We hope that our concerns will help advance the discussion of this study.

## EDITOR'S NOTE

**Ed. Note:** The authors of the original article declined to respond.
